# Diagnosing B-cell acute lymphoblastic leukemia in 2 pediatric patients with recent SARS-CoV-2 infection

**DOI:** 10.1177/2632010X241278180

**Published:** 2024-09-06

**Authors:** Anupam Mitra, Alexander Ladenheim, Ananya Datta-Mitra, Kaitlyn Lauren Honeychurch, Denis M Dwyre, John Paul Graff

**Affiliations:** 1 Department of Pathology and Laboratory Medicine, University of California Davis Medical Center, Sacramento, CA, USA; 2 Department of Pediatrics, University of California Davis Medical Center, Sacramento, CA, USA

**Keywords:** Acute leukemia, lymphoblastic leukemia, COVID-19, fluorescence in situ hybridization, pre-leukemic genes

## Abstract

COVID-19 infection is still a mystery in terms of its long-term effect on health and its consequences on hematological disorders. Prior studies including ours have shown the abnormal changes in hematopoietic cells in COVID-19 patients. In this article, we are presenting 2 cases of pediatric B-lymphoblastic leukemia (B-ALL) with a previous history of COVID-19 infection. The first case describes a 22-month-old boy presenting with lymphadenopathy, neutropenia, and anemia with concurrent COVID-19 infection without any evidence of a hematolymphoid neoplasm as per bone marrow and lymph node biopsy. However, he presented after 2 months with bone marrow biopsy confirming B-ALL. The second case is that of a 4-year-old girl presenting with B-ALL who has had asymptomatic COVID-19 infection 5 months before this current presentation. Both the cases had complete resolution of COVID-19 infection during the time of presentation with acute leukemia. There were notably 2 rare findings along the course of the patients’ illnesses. First, the unusual plasmacytosis in the marrow during active COVID-19 infection in the first patient and the second, is predilection of development of B-ALL following COVID-19. In both the cases the fluorescence in situ hybridization (FISH) studies showed pathologic alteration of the *RUNX1* gene. Overall, there are no literature to support a causal association between acute B-ALL and COVID-19. The diagnosis of B-ALL in these patients after COVID-19 infection may be totally unrelated. However, if we consider Greaves proposed 2-hit model for childhood acute leukemia, that an infectious agent can precipitate development of B-ALL in a genetically susceptible individual. Alteration of the RUNX1 gene in both the patients, opens a door for further exploration of the “second-hit” hypothesis regarding an infectious agent precipitating development of B-ALL in a genetically susceptible individual.

## Background

Since its inception in 2019, coronavirus disease 2019 (COVID-19) rapidly became a pandemic affecting almost all the countries of the world. Prior studies including ours^1^ showed the effect of COVID-19 in hematopoietic cells causing lymphopenia, leukocytosis, neutrophilia, thrombocytopenia, and leukoerythroblastosis. However, effect of COVID-19 and hematologic malignancies has not been reported widely. In this article, we are presenting 2 unique cases of B-lymphoblastic leukemia (B-ALL) in pediatric patients with a previous history of COVID-19 infection. Both the cases had complete resolution of COVID-19 infection during the time of presentation with acute leukemia.

## Case Presentation

*Case 1:* A 22-month-old male up to date on vaccination with a 7-day history of intermittent fever, rhinorrhea, cough, poor appetite, cervical lymphadenopathy, and fatigue presented to an outside hospital. He was positive for SARS-CoV-2 and was noted to be neutropenic and anemic and transferred to our hospital.

**Figure 1.**
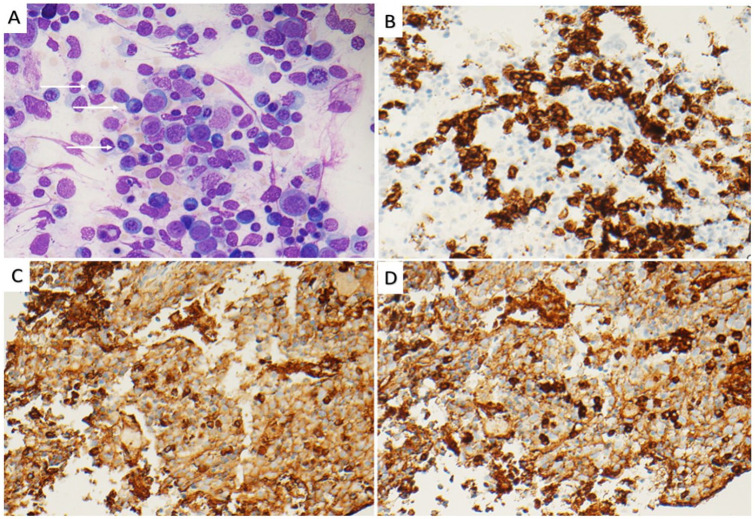
(A) Wright-Giemsa stained of bone marrow aspirate smear showing intact hematopoiesis with plasmacytosis (white arrows; 50x). Plasma cells are highlighted by CD138 IHC 40x magnification (B). The plasma cells are polytypic for Kappa-IHC (C) and Lambda-IHC (D), 40x magnification.

On arrival, he was tachypneic, tachycardic, and febrile (39.1°C), but otherwise hemodynamically stable with an oxygen saturation of 98% to 100%. The physical exam findings were significant for cervical lymphadenopathy. A complete blood count (CBC) showed leukopenia (1.4 × 10^3^/mL) with marked neutropenia (absolute neutrophil count of 0), normocytic anemia (hemoglobin 7.1 g/dL) with mild anisopoikilocytosis, and thrombocytosis (435 × 10^3^/mL). There is an increase in C-reactive protein (CRP, 20.1 mg/dL) and ferritin (459 ng/mL, reference range 22-322 ng/mL). A biopsy of the marrow and the enlarged lymph node was done due to the lack of clinical improvement and persistence of anemia and neutropenia. The bone marrow aspirate showed normal trilineal hematopoiesis and a marked increase in polytypic plasma cells (~31%; Figure 1A-D). There was no increase in blasts. A lymph node biopsy did not reveal any pathology. The patient was treated conservatively and got discharged with a negative COVID-19 test.

**Figure 2.**
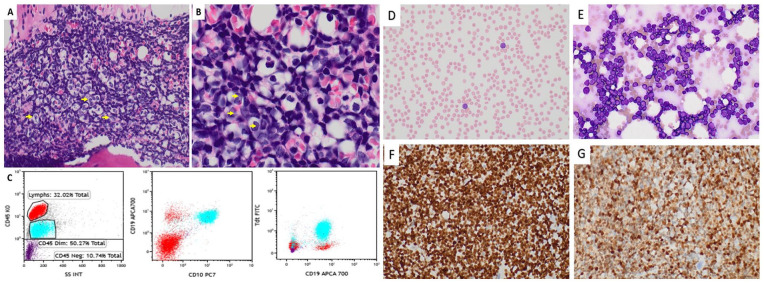
Slides from the trephine bone marrow core biopsy (case 1). (A) 40x magnification of H&E demonstrating increased lymphoblasts as noted by yellow arrows 100x oil immersion magnification (B). (C) Flow cytometry images showing the blasts are CD45 dim, expressing CD19, CD10, and TDT. Peripheral blood and bone marrow of case 2. (D) peripheral blood smear 40X showing pancytopenia with circulating blasts. (E) touch prep 40x showing increased sheets of blasts highlighted by Pax-5 (F) 40X and TDT (G) 40X.

Two months later, the patient returned with febrile seizure and was positive for respiratory syncytial viral (RSV) infection and was managed symptomatically and discharged. At that time, he was negative for flu and SARS-CoV-2. He again returned after 4 days with fever and cervical lymphadenopathy. An infectious work-up this admission was negative. CBC showed a new finding of blasts in addition to persistent neutropenia. A second bone marrow biopsy with flow cytometry was performed revealing B-ALL with 54% B-lymphoblasts (Figure 2A-C). No plasmacytosis was noted.

*Case 2:* This is a 4-year-old girl presenting with daily fever (101°F-103°F) for 3 weeks along with knee pain, back pain, decreased appetite, decreased activities and fatigue. She was treated empirically with antibiotics for urinary tract infection, however with no relief and was brought to the ER. She did not have any significant past medical history apart from asymptomatic COVID-19 infection 5 months back with complete recovery. She was up-to-date on her vaccinations and was unvaccinated for COVID-19. On physical examination she had fever (100.8°F), conjunctival and skin palor, palpable hepatomegaly with no splenomegaly and no abdominal tenderness. Her other exam findings were normal and there was no palbable lymphadenopathy. The CBC showed normocytic anemia (Hb: 8.3 g/dL), leukopenia (0.6 × 10^3^/mL) with marked neutropenia (absolute neutrophil count of 0.1) thrombocytopenia (23 K/mm^3^), and circulating blasts (Figure 2D). A bone marrow examination was performed showing aplastic trilineage hematopoeisis with increased blasts (Figure 2E) with a B-ALL immunophenotype, PAX5+/TDT+ (Figure 2F and G)/CD34+ (not shown).

Both the cases were started induction chemotherapy with vincristine, dexamethasone, peg-asparaginase, and intrathecal cytarabine/methotrexate.

## Discussion

In this case series, we noticed 2 unique findings along the course of the patients’ illnesses. First, the unusual plasmacytosis in the marrow during active COVID-19 infection in the first patient and the second, is predilection of development of B-ALL following COVID-19. The peripheral blood findings in the setting of an acute COVID-19 infection as described previously,^1-3^ include lymphocytopenia, neutropenia, left shifted neutrophilia, and leukoerythroblastosis. Few bone marrow studies in COVID-19 positive patients have been reported, mostly from autopsy studies.^4-6^ Hemophagocytosis is the most common finding reported in the bone marrow of these patients, who presumably had more severe COVID-19 complications. Two studies demonstrated an increased bone marrow plasma cells in a series of COVID-19 autopsies.^4,5^ However, the authors did not quantify the degree of plasmacytosis nor were the plasma cells assessed for clonality. In our first case, no hemophagocytosis was noted on the bone marrow aspirate, and a prominent bone marrow polytypic plasmacytosis was seen, an especially unusual finding in a child.

Limited marrow examination studies in infected patients, have failed to definitively attribute the presence of increased plasma cells in this patient to COVID-19 infection. However, the plasmacytosis, elevated CRP, increased ferritin, and IgA and IgE hypergammaglobulinemia are suggestive of a hyperinflammatory reaction. Hemophagocytosis and associated cytokine storm-like clinical presentation are the more commonly described hyperinflammatory reaction in severe COVID-19 infection.^7^ In a similar context, prior studies have showed the presence of excess number of plasma cells in bronchoalveolar lavage fluid of patients with severe COVID-19.^8,9^

It has also been shown that reactive plasmacytosis can be seen in certain viral infections which can even mimic a plasma cell neoplasm in their initial presentation.^10-12^ A recent study showed that presence of circulating peripheral plasma cells in the context of COVID-19 infection is associated with a lower mortality rate.^13^ In our case, we did not find any circulating plasma cells in the peripheral blood.

Moreover, there is no known association between acute B-ALL and COVID-19, and COVID-19 infection precipitating the development of B-ALL has not been described in the literature. The diagnosis of B-ALL in our 2 patients is a second separate malady and may be unrelated to the patients’ prior COVID-19 infections. It has been hypothesized, however, that an infectious agent can precipitate development of B-ALL in a genetically susceptible individual.^14-16^ The most common translocation occurring in pediatric B-ALL is *ETV6-RUNX1* [t(12;21)].^15,16^ In this context, Greaves proposed a 2-hit model for childhood acute leukemia. Briefly, as per this model, the “first-hit” happens in utero with the generation of a preleukemia clone which harbors leukemia-associated genetic changes. A subsequent “second-hit” occurs during early childhood possibly by infectious agents such as viruses, which ultimately favors the proliferation of the abnormal clones leading to development of acute leukemia.^14,15^ In their letter to the editor, Taub et al^17^ have also suggested a possibility that control of COVID-19 infection in the pediatric population through isolation might reduce the effect of the “second-hit” leading to B-ALL. In response to their hypothesis Greaves proposed to further investigate the beneficial effect of COVID-19 isolation, with screening for viral antibodies in cases of B-ALL diagnosed over 2020 and beyond and compare the changes in the incidence rates of B-ALL in areas with widely differing COVID-19 incidence and areas with no isolation policies in place.^18^ A study in Germany found out that there is a significant increase in incidence of B-ALL in 2 to 6-year-olds in 2020 as compared to the previous years.^19^ However, they could not substantiate their data in 7- to 14-year-old age where the same infection control measures were applied. Our first patient had COVID-19 infection followed by a RSV infection 2 months later. Either of these infections or both could potentially contribute as a “second hit” and played a role in developing B-ALL. Our first patient’s cytogenetics study showed a complex abnormal karyotype with trisomies 1,10, 11, 12, 17, and 22 and tetrasomies 4, 8, and 21. FISH study showed pathologic alterations in the known pre-leukemic genes such as *RUNX1* and/or *ETV6*. The second patient’s FISH detected trisomy 4 8, 14, 10, and 17, and 4 copies of *RUNX1* (at 21q22, corresponding with trisomy 21), in addition and deletion of *CDKN2A*. These observations together may suggest the role of COVID-19 as a “second-hit” in development of B-ALL, but this is a very preliminary observation and based on only 2 cases, and further observations are required to explore this association.

To the best of our knowledge, our case report is the first to show marked polyclonal plasmacytosis in an alive COVID-19 patient. This report also found development of B-ALL in patients following COVID-19 infection similar to previous published case reports.^20-21^ The development of B-ALL in these patients may or may not be associated with prior COVID-19 infection but keeping in mind the 2-hit model of B-ALL, this merits further investigation to characterize their role.
